# The palliative care needs of adults with intellectual disabilities and their access to palliative care services: A systematic review

**DOI:** 10.1177/0269216320932774

**Published:** 2020-06-17

**Authors:** Emily Adam, Katherine E Sleeman, Sarah Brearley, Katherine Hunt, Irene Tuffrey-Wijne

**Affiliations:** 1 Cicely Saunders Institute of Palliative Care and Rehabilitation, King’s College London, London, UK; 2 Faculty of Health and Medicine, Lancaster University, Lancaster, UK; 3 Faculty of Health Sciences, University of Southampton, Southampton, UK; 4 Faculty of Health, Social Care & Education, Kingston University and St George’s, University of London, London, UK

**Keywords:** Palliative care, health services needs and demand, intellectual disability, health services accessibility

## Abstract

**Background:**

There is evidence that people with intellectual disabilities experience healthcare inequalities, including access to specialist palliative care, but to date, there has not been a systematic review of empirical evidence.

**Aim:**

To identify the palliative care needs of adults with intellectual disabilities and the barriers and facilitators they face in accessing palliative care.

**Design:**

Systematic review using a narrative synthesis approach (International prospective register of systematic reviews (PROSPERO) registration number: CRD42019138974).

**Data sources:**

Five databases were searched in June 2019 (MEDLINE, Embase, PsycINFO, the Cochrane library and CINAHL) along with hand searches and a search of the grey literature. All study designs were included.

**Results:**

A total of 52 studies were identified, all of which were conducted in high-income countries, the majority in the United Kingdom (*n* = 28). From a total of 2970 participants across all studies, only 1% were people with intellectual disabilities and 1.3% were family members; the majority (97%) were health/social care professionals. Identified needs included physical needs, psychosocial and spiritual needs, and information and communication needs. Barriers and facilitators were associated with education (e.g. staff knowledge, training and experience), communication (e.g. staff skill in assessing and addressing needs of people with communication difficulties), collaboration (e.g. importance of sustained multidisciplinary approach) and health and social care delivery (e.g. staffing levels, funding and management support).

**Conclusion:**

This review highlights the specific problems in providing equitable palliative care for adults with intellectual disabilities, but there is a lack of research into strategies to improve practice. This should be prioritised using methods that include people with intellectual disabilities and families.

**What is already known about the topic?**

- Adults with intellectual disabilities experience health care inequalities and are less likely to have access to palliative care.
- The specific palliative care needs of people with intellectual disabilities are poorly understood.
- Guidance and policy in this field is mostly built upon theoretical and anecdotal evidence with a very limited empirical knowledge base.

**What this paper adds?**

- While the palliative care needs of adults with intellectual disabilities mirror those of the general population, there are specific and complex challenges associated with these individuals.
- Adults with intellectual disabilities face multiple barriers to accessing palliative care.
- There is a paucity of high-quality research in this field and people with intellectual disabilities themselves, and their families, are not represented through the currently available evidence.

**Implications for practice, theory or policy**

- Addressing the inequalities in accessing palliative care for people with intellectual disabilities should be an urgent international priority.
- Examples of good practice and promising initiatives identified in this review need to be supported by good quality research, embedded in national policy and adequately funded.
- Future research should focus on developing measurable outcomes specifically related to people with intellectual disabilities to allow for large-scale interventional studies that demonstrate these initiatives are effective and worthwhile.

## Introduction

The global prevalence of intellectual disability is estimated at approximately 1%.^1^ Improved health and social care means this population is expanding.^1^ While there are various definitions of intellectual disability, there is international consensus that it is present when the following three criteria are met: a significantly reduced ability to understand new or complex information and to learn and apply new skills (impaired intelligence); a reduced ability to cope independently (impaired social functioning); and beginning before adulthood, with a lasting effect on development.^2–4^ In the United Kingdom, people with intellectual disabilities make up around 1.5 million people, and this is expected to rise by 1.1% annually, with those aged over 60 years set to increase substantially.^5^ With an ageing population comes a rising prevalence of chronic and age-related illness, and subsequently an increased need for palliative and end-of-life care.^5–8^

Despite this trend, people with intellectual disabilities die approximately 25 years sooner than the general population.^9^ Evidence from the World Health Organization (WHO) atlas of global resources for people with intellectual disabilities reveals that people with intellectual disabilities are often denied their right to health care, even in countries with a relatively high standard of living.^10,11^ Worldwide, there is a paucity of documentation, information or epidemiological data about this population.^10^ As such, it is challenging to fully appreciate the prevalence and impact of intellectual disabilities on health care needs, and the associated barriers and facilitators experienced. Investigations into the deaths of people with learning disabilities in the United Kingdom^9,12,13^ have identified institutional discrimination and considerable evidence of health care inequalities contributing to avoidable excess mortality. In addition, people with intellectual disabilities were less likely to have access to specialist palliative care services and received less opioid analgesia in their final illness than people without intellectual disabilities.^13^ The independent regulator of health and social care in England (the Care Quality Commission (CQC)) found that exclusion of people with intellectual disabilities from wider health services was a significant barrier to care at end of life.^14^

Existing guidance to improve palliative and end-of-life care for people with learning disabilities is mostly based on theoretical or anecdotal evidence, expert opinion and case reports.^6,14,15^ Guidance from the National Institute for Health and Care Excellence (NICE) in the United Kingdom regarding older people with intellectual disabilities^16^ was based on a systematic review which identified only two studies reflecting the views of people with intellectual disabilities and their families. In order to understand more about this gap in knowledge, and about the best direction for further research in this area, it is essential to examine in detail what is known about the inequalities faced by this population. The aim of this systematic review was to identify the palliative care needs of adults with intellectual disabilities and the barriers and facilitators this population face in accessing palliative care.

## Methods

The protocol for this systematic review was registered in the PROSPERO database^17^ (CRD42019138974) and is reported according to the Preferred Reporting Items for Systematic Reviews and Meta-Analyses (PRISMA) guidelines.^18^

### Review questions

What are the palliative care needs of adults with intellectual disabilities?
What are the barriers and facilitators this population face in accessing palliative care?

### Search strategy

MEDLINE, Embase, PsycINFO, the Cochrane library and CINAHL were searched in June 2019. MeSH terms and key words, guided by previously published systematic reviews,^19–21^ were combined with a pre-defined palliative care filter^22^ to form the search strategy (Supplemental Appendix 1). Hand searching included two intellectual disability specific journals: *Journal of Applied Research in Intellectual Disabilities* (*JARID*) and *Journal of Intellectual Disability Research* (*JIDR*). These were considered to be the most relevant journals in the field of intellectual disability. They were searched online, without any date restrictions, to check for articles that may not have been captured in the database search. Reference lists of key publications^6,9,13–15,23,24^ and of identified studies were also reviewed. The grey literature was searched online using OpenGrey^25^ and CareSearch.^26^

### Inclusion and exclusion criteria

An inclusive approach was adopted given the paucity of research in this field. Multiple study designs and all health care settings were included and no geographical or date limitations applied. Table 1 details inclusion and exclusion criteria.

**Table 1.** Eligibility criteria.

Inclusion criteria	
Population	• Studies including adult participants aged 18 and over with intellectual disabilities and a ‘life-threatening illness’ as per the WHO definition of palliative care.^27^ • Studies including participants who are carers/relatives or health/social care staff caring for this population.
Setting	• Home, hospital, hospice, nursing/residential home, outpatient and primary care/community. • Worldwide.
Outcomes	• Any outcomes relating to palliative care needs of adults with intellectual disabilities. • Any outcomes describing barriers and/or facilitators to accessing generalist or specialist palliative care.
Study designs	• Qualitative and quantitative research methods. • Experimental study designs: randomised controlled trials (RCTs) and quasi-experimental. • Observational study designs: cross-sectional, cohort and case-control. • Prospective and retrospective designs. • Literature reviews and systematic reviews. • Case series and case reports.
Exclusion criteria	
• Discussion and opinion papers, conference abstracts, editorials, letters, comments and guidelines. • Non-English articles where translation cannot be achieved.	

WHO: World Health Organization.

### Study selection

**Figure 1.**
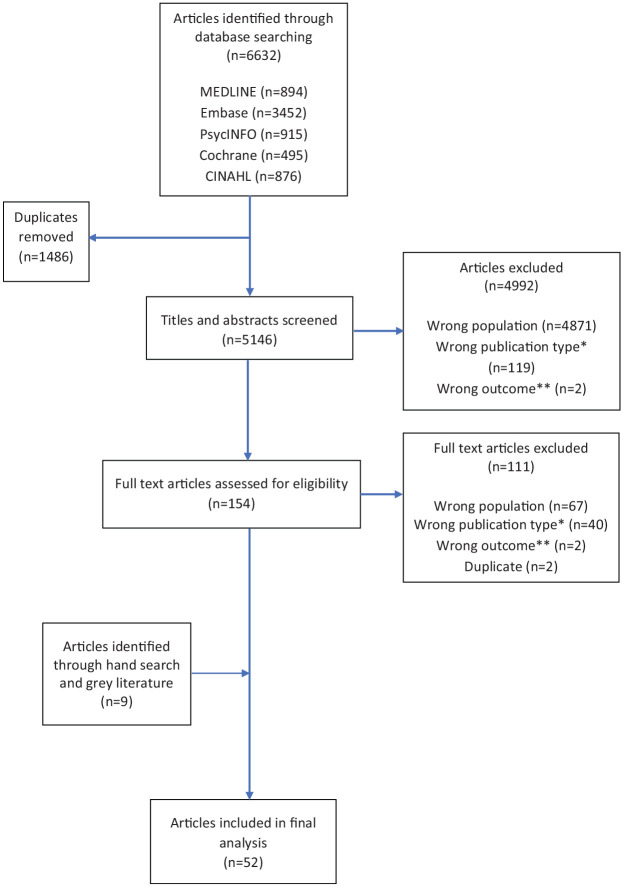
Screening process based on the PRISMA flow diagram.^18^ *Wrong publication: fell into one of the following categories in the exclusion criteria: ‘discussion and opinion papers, conference abstracts, editorials, letters, comments, guidelines’. **Wrong outcome: did not include ‘Any outcomes relating to palliative care needs of adults with intellectual disabilities’ or ‘Any outcomes describing barriers and/or facilitators to accessing generalist or specialist palliative care’ as listed in the inclusion criteria.

The electronic searches identified 6632 articles. Following removal of duplicates, titles and abstracts were reviewed to assess if they merited full text analysis. A second researcher (I.T.-W.) reviewed a sub-set of 100 randomly selected titles and abstracts to check for inter-rater agreement with the primary researcher (E.A.). Any disagreement was resolved with a third researcher (K.E.S.). A kappa value of 0.67^28,29^ demonstrated ‘substantial’ agreement. Analysis of the full text was then undertaken by E.A. Endnote^30^ and Rayyan^31^ were used to manage the selection process. A total of 52 articles were included for final analysis. Figure 1 details the selection process.

### Data extraction

Data were extracted using a Microsoft Excel^32^ template (Supplemental Appendix 2) developed with guidance from the Cochrane ‘Checklist of items to consider in data collection or data extraction’.^33^

### Grading of quality

In order to evaluate the quality of the evidence included in the review, the articles were assessed using Hawker et al’.s^34^ critical appraisal tool (Supplemental Appendix 3). This was designed specifically to assess research conducted using different paradigms.^34^ It consists of nine questions, each of which are scored on a 4-point scale from very poor (1 point) to good (4 points). Based on a scoring system adapted by Voss et al.,^20^ total scores of 18 or less were defined as poor, 19–27 as moderate and above 27 as good (Supplemental Appendix 4).

### Data analysis

Information was collected for any outcomes relating to the palliative care needs of adults with intellectual disabilities and/or facilitators and/or barriers to accessing palliative care. Due to the heterogeneity of the studies, narrative methods were employed to synthesise the data. This encompassed the generation of themes, which was supported by the qualitative data analysis software NVivo.^35^ This narrative synthesis approach involved collating study findings into a textual narrative, along with tables and graphs. It was considered an appropriate choice of method, being suited to both quantitative and qualitative data and where statistical synthesis is not possible.^36,37^

## Results

### Study characteristics

All studies were conducted in high-income countries, with more than 50% conducted in the United Kingdom (Table 2).

**Table 2.** Geographical location of research.

Country	Number of articles
United Kingdom	28
Netherlands	6
United States	6
Ireland	4
Australia	3
Multiple European countries	3
Canada	1
New Zealand	1
Total	52

Qualitative methods were the most commonly used (*n* = 17). There were 12 mixed method studies, 12 literature reviews, 10 studies with quantitative methods and 1 systematic review. There were no randomised controlled trials (RCTs). According to Hawker et al’.s checklist,^34^ four articles were graded ‘poor’, 25 were ‘moderate’ and 23 were ‘good’ (Supplemental Appendix 4).

The studies covered a range of health and social care settings including home, hospice, hospital, community, residential and nursing homes. Many studies did not describe the setting (*n* = 24). For those that did (*n* = 28), most involved participants based in community or residential settings (*n* = 16).

**Figure 2.**
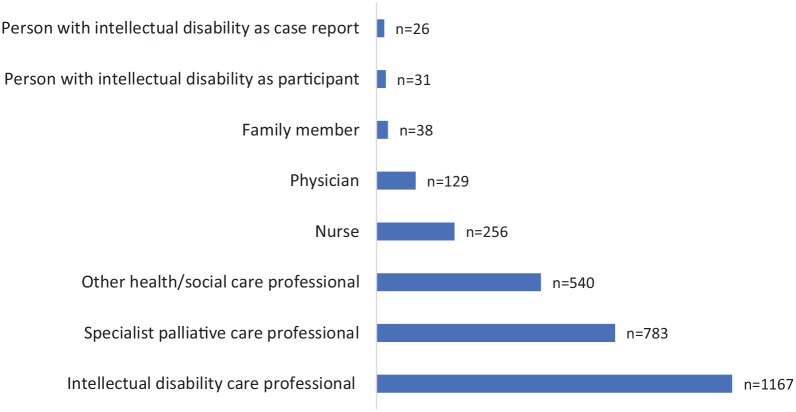
Participants across studies.

There were 2970 participants included across the 52 studies. The majority of participants were health or social care professionals (*n* = 2875, 96.8%). There were more intellectual disability professionals (*n* = 1167, 39.3%) than there were specialist palliative care professionals (*n* = 783, 26.4%) and more nurses (*n* = 256, 8.6%) than physicians (*n* = 129, 4.3%). There were 38 family members (1.3%) and 31 people with intellectual disabilities and a life-threatening illness (1%) acting as participants themselves (Figure 2).

## Qualitative synthesis

The data were synthesised according to needs, barriers and facilitators.

### Needs

Information relating to the palliative care needs of people with intellectual disabilities was identified in 32 studies. With the exception of one paper,^38^ all of these studies reflected the perspectives of health care professionals or relatives. Three themes were identified: (1) physical needs, (2) psychosocial and spiritual needs and (3) information and communication needs.

#### Physical needs

The most common physical need identified by staff was pain management. Many health care professionals described the challenge of symptoms being expressed as objectively observable signs or behaviours such as irritability, inactivity, loss of appetite and sleep disturbance, rather than spontaneous complaints.^39^ Other symptoms requiring attention included nausea, vomiting, fatigue,^40^ shortness of breath, constipation, delirium,^41^ urinary incontinence and oral thrush.^42^ Monitoring of hydration and nutrition was also important.^42–46^ Many patients experienced declining mobility and needed physiotherapy.^42,46,47^ Physical needs also involved wound care,^41,48^ pressure area care^44^ and personal care such as washing.^49^

#### Psychosocial and spiritual needs

The importance of family and the need for a social network was expressed widely.^47,50,51^ A study involving interviews with health professionals and carers highlighted that people with intellectual disabilities at the end of life need to be surrounded by people that know them well and can advocate for them.^46^ Socialising,^52^ friendships^3^ and human contact^53^ are important. People with intellectual disabilities who are dying require continuation of safe routines, treasured activities and important relationships.^38^ One study also highlighted the need to be occupied.^51^ Many studies recognised the importance of creating a familiar and predictable environment.^54^ For many this involved provision of care in the person’s home for as long as possible.^42,44,46,55^ A change in environment or unfamiliar faces may cause distress and hinder communication.^1,42,49^

People with intellectual disabilities and a life-limiting illness experience fear and anxiety^38,49^ particularly when receiving bad news^40^ or having difficulty understanding medical information.^38^ Some studies spoke of grief and loss,^45^ non-verbal expression of grief^56^ and the recognition of complicated grief,^3,56^ and endorsed the need for appropriately tailored counselling services.^3^ There is also evidence that many people with intellectual disabilities have additional mental health problems which require increased support measures.^49^ Spiritual needs were identified, but not explored in detail.^46,47,52^ The need for culturally appropriate care was also highlighted.^45,46,48^

#### Information and communication needs

People with intellectual disabilities are often not provided with information in an accessible format.^50,57^ This is important in enabling them to understand their diagnosis, prognosis and the symptomatic course of their illness.^1,58^ It is the responsibility of health care professionals to consider communication differences and the difficulty people with intellectual disabilities may have in understanding abstract concepts.^54^ People with intellectual disabilities often need help to express their views and participate in decision-making.^59^ They need honest communication and opportunities to make choices.^46,60,61^ This is also important for advance care planning, with several papers placing emphasis on the need to discuss, document and respect preferred place of care and death, which tended to be home.^1,43,46,62,63^

### Barriers and facilitators

**Figure 3.**
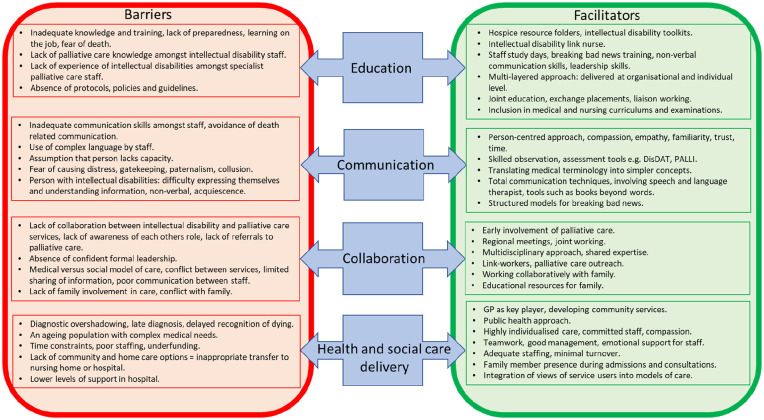
Conceptual model of barriers and facilitators to accessing palliative care for adults with intellectual disabilities.

Many factors acted as barriers as well as facilitators to providing palliative care to people with intellectual disabilities, depending on whether they were present or absent. Four themes were identified: (1) education, (2) communication, (3) collaboration and (4) health and social care delivery. A conceptual model of barriers and facilitators was developed from these themes (Figure 3).

#### Education

##### Education – barriers

Inadequate education was the most widely reported barrier. Particularly evident was the lack of experience, preparation and training in delivering end-of-life care among staff looking after people with intellectual disabilities in community or residential settings.^46,48,50,62,64–70^ Lack of preparedness for seeing residents entering the dying phase of their lives hindered engagement with palliative care services.^68^ A survey exploring the educational needs of intellectual disability care practitioners revealed poor understanding of diagnosis and causation of death, which raised concerns about their ability to recognise a dying patient.^48^ These issues are compounded by carers working in isolated community settings without access to guidelines.^42^ It was often standard practice for support workers to be on their own with residents when they were dying^68^ and staff that spent the most time with patients were often the least trained.^54^

The data also reflected a need for specialist palliative care professionals to improve their knowledge and experience in managing patients with intellectual disabilities.^8,49,53,54,65,71–73^ In addition, several studies highlighted the need to educate registered nurses in both hospital and community settings in caring for patients with intellectual disabilities at the end of life.^8,44,61,74–76^

Inadequate training can heighten communication fears,^67^ lead to lack of confidence among staff^51^ and elevated levels of stress.^45^ An intellectual disability support worker commented in a focus group discussion that there was a tendency of staff in hospital settings to put patients with intellectual disabilities and palliative care needs into the ‘too-hard basket’.^8^

Contributing to this knowledge gap is the absence of formal protocols, policies and guidelines regarding end-of-life care for people with intellectual disabilities.^45,55,62^ A study examining guidelines in 11 European countries suggested that European national palliative care guidelines do not meet the needs of people with intellectual disabilities.^77^ Lack of organisational policy leads to inconsistent practice across settings and a postcode lottery whereby end-of-life care outcomes are often determined by individual staff.^62,68,78^

##### Education – facilitators

Proposed educational incentives included a hospice resource folder containing information on local intellectual disability services, an intellectual disability toolkit designed to support hospital professionals and recruitment of a hospice intellectual disability link nurse to encourage integrated learning.^79^ Some studies suggest specific areas to focus on such as breaking bad news training for staff in intellectual disability settings,^40^ advanced training on recognition of non-verbal signs of pain^72^ and leadership skills.^62^ There is evidence that education in the form of a study day for paid carers can lead to improved knowledge and increased awareness about end-of-life care.^67^ A multi-layered approach to learning, delivered at both the individual and organisational level, contributed to successful implementation of the ‘Steps to Success Palliative Care Programme’ for people with intellectual disabilities living in residential care homes.^59^

Professionals working within intellectual disability and palliative care services expressed a desire to learn from each other through joint education, exchange placements and liaison working.^71^ Small group discussion between peers^70^ and hearing the perspectives of people with intellectual disabilities themselves were also effective educational techniques.^70^ Wider approaches are the inclusion of palliative care for people with intellectual disabilities as a core component in medical and nursing curriculums^48^ and examinations.^1^

#### Communication

##### Communication – barriers

Many studies reported inadequate communication skills among staff working with people with intellectual disabilities.^44,51,53,55,67,69,79,80^ Fear of initiating conversations about death and lack of experience in breaking bad news were widely reported issues,^61,67,70^ with a tendency in intellectual disability settings to keep things positive.^70^ Staff were also concerned that they may cause distress to the person with intellectual disabilities.^70^ Health care professionals may use complex language^47^ and fail to recognise the difficulty people with intellectual disabilities can have in understanding abstract concepts relating to death.^56,57^ An assumption that the person with intellectual disabilities lacks capacity and cannot provide informed consent^58^ leads to an over-reliance on carers or relatives as communication proxies,^51,56,73^ facilitates information gatekeeping and prevents open discussion.^38,51,73,79^ This paternalistic approach presents a barrier to effective palliative care.^46,49,51,61^

Communication barriers also relate to the impaired ability of a person with intellectual disabilities to express themselves and understand information.^51,53,55,69,80^ People with intellectual disabilities who are non-verbal present a particular challenge for staff and carers.^55,60,74,79,81^ Symptom assessment is difficult, and there may be a diversity in signals that lead to recognition of the dying phase.^82^ Acquiescence also presents a challenge for health care professionals, who may not recognise that people with intellectual disabilities are unlikely to question treatment decisions.^38^ Difficulties confirming understanding, ascertaining information requirements and establishing wishes^45^ can result in failure to involve people with intellectual disabilities in the decision-making process.^1,51,81^ This can lead to conflict and uncertainty when the person’s health deteriorates.^83^ A UK survey describing end-of-life care outcomes for adults with intellectual disabilities found that few individuals had their end-of-life preferences recorded and the majority were not aware they were going to die.^63^

##### Communication – facilitators

A person-centred approach incorporating compassion and empathy are important when caring for people with intellectual disabilities who have difficulty communicating.^7,67,69^ Taking time to build relationships, gain trust and confirm understanding can facilitate effective communication.^1,43,49,51,60,73^ As people with intellectual disabilities are likely to take longer to express themselves and may rely on communication tools, health care professionals should allocate more time for consultations.^39,60^ This is particularly important when talking about death and dying.^61^ Continuity of care^43,45,73^ and involvement of a family member can also aid communication.^3,39,41,56,58^ Helping people with intellectual disabilities to understand and cope with bad news requires building of knowledge gradually over time and support from the person’s family and professional network.^57^ Translating medical terminology into simpler concepts maximises involvement in decision-making.^43^ Involving speech and language therapists is another approach.^46,55^ Pictorial books designed to help people with intellectual disabilities understand and discuss terminal illness can be helpful.^56,60^ Structured models for breaking bad news to people with intellectual disabilities have also been developed.^56,57^

Tools to aid assessment of physical symptoms include the ‘DisDAT’ (Distress Assessment Tool),^39,56,58,67^ developed for people with severe communication problems including those with intellectual disabilities, the ‘REPOS’ (Rotterdam Elderly Pain Observation Scale)^41^ and the Abbey scale.^56^ The ‘PALLI’ (PALliative care: Learning to Identify in people with intellectual disabilities) is a tool for use by proxies.^84^

#### Collaboration

##### Collaboration – barriers

Lack of collaboration between services was a widely reported barrier. Most often this was between intellectual disability and specialist palliative care services.^44,53,62,65,71,72^ Evidence suggests poor referral rates for patients with intellectual disabilities to specialist palliative care services,^54,65,71^ with few patients receiving dual hospice and intellectual disability care.^72^ There is a deficiency of established relationships between intellectual disability and palliative care services^72^ encompassing a poor understanding of each other’s role, what the service is providing and how it is run.^54,65^ For example, one paper described an intellectual disability care home manager who did not know how to access the palliative care team.^42^ Another issue is the medical versus social model of care.^55,71^ Carers familiar with the social care model may neglect the physical aspects of care for the dying.^48^ Interviews with intellectual disability and specialist palliative care professionals revealed mistrust between services or conflict regarding ownership of the patient.^71^ Limited sharing of information between services and poor referrals can lead to inadequate knowledge of the patient.^42,44,61^ A view that patients require specialist intellectual disability services leads to exclusion from general palliative care services^65^ and a reluctance of intellectual disability services to acknowledge death can mean patients who are dying often remain hidden.^46^

Family members are often expected to make complex ethical decisions,^83^ and lack of understanding regarding focus of care in advanced illness^44,56^ can lead to limited cooperation with palliative care services.^44^ Conflicts between staff and surrogate decision makers have been cited as a common barrier to hospice care.^80^ One study identified several nurses who were barred from visiting a client by family members who feared they would disclose a poor prognosis.^57^

##### Collaboration – facilitators

Collaborative working between palliative care services, intellectual disability services and carers was an effective way to deliver care to people with intellectual disabilities.^43,44,49,51,65,68,79^ Earlier involvement of palliative care builds familiarity and trust between staff and services.^56^ Incentives such as regional meetings, joint working or shadowing in both clinical areas can be effective.^71^ A mixed methods study described the United Kingdom’s first specialist palliative care home for older people with intellectual disabilities, demonstrating positive results for quality of life.^7^

Key to effective collaboration is a multidisciplinary approach allowing shared expertise between intellectual disability, specialist palliative care, hospital services, community teams and GPs.^40,46,52^ Specialist palliative care professionals found that liaising with intellectual disability professionals who knew the patient well was helpful around issues of mental capacity and consent.^49^ Link workers acting as conduits between palliative care and intellectual disability services are also helpful.^52–54,65^ Working collaboratively with the family should encompass recognition that they know the person best, building trust, sharing information, involving them in decision-making and supporting them emotionally.^44,45,68,79^

#### Health and social care delivery

##### Health and social care delivery – barriers

Diagnostic overshadowing is a prevalent issue^8,38,49^ resulting in late diagnosis of terminal illness^54^ and delayed recognition of dying.^82^ Compliance with care, examination or prescribed medication may be a challenge among people with intellectual disabilities^54,61^ which can also compromise identification and management of symptoms. Some doctors declined to take on patients with intellectual disabilities at the end of life because they lacked the time to manage their complex medical issues.^8^ People with intellectual disabilities often have unpredictable clinical trajectories making it difficult to prognosticate,^56^ and an ageing intellectual disability population brings changing health care needs and disease profiles.^81^ The authors also observed that time constraints,^71^ inadequate staffing levels^62,64,69^ and underfunding^8,46,78,80^ were barriers to supporting the additional health care needs of people with intellectual disabilities at the end of life.

An ageing intellectual disability population means parent caregivers are increasingly elderly, and for people with intellectual disabilities living at home, lack of home care options means they require transfer to long-term facilities when their health deteriorates and their parents can no longer cope.^49,53^ Given the short-term nature of hospices, many people with intellectual disabilities are misplaced in nursing homes for people much older than themselves, which lack the expertise to meet their needs.^7,47^ There are few nursing homes that have this expertise.^7,49^ Intellectual disability staff resistance to provide end-of-life care at home may also prompt transfer to hospital or a nursing home when their client’s health deteriorates.^8^

##### Health and social care delivery – facilitators

Several studies have highlighted the role of the GP as a key player in identification of need and coordinating referrals for people with intellectual disabilities.^43,46,71^ Developing community-based services with input from GPs and district nurses will support people with intellectual disabilities living at home and allow them to die there.^8,43,62,64,74,78^

Delivery of palliative care to this population is often dependent on committed staff who are willing to work beyond their call of duty.^7,62,78^ Delivery of highly individualised care requires teamwork, empathy and enthusiasm.^44,62,69,78^ Many studies highlighted the value of good management and support for staff within their own organisation.^7,45,62,68^ This should encompass emotional and bereavement support.^38,46,61,67,68,71^ Building resilience and empowering the workforce enables them to deal with grief and in turn support the patient.^57,62,70,71^ In organisations where managers provided positive role modelling by talking about death and dying, junior staff were more likely to feel comfortable discussing these topics.^70^ Adequate staffing and minimal staff turnover were also important to deliver effective and sustainable palliative care to this population.^7,68,78^

Simple practical adjustments that help people with intellectual disabilities engage with palliative care services include allowing the presence of a family member or keyworker during hospital admissions, consultations and investigations^42,43,46^ and visits to hospital or treatment units beforehand.^40^ Good practice also includes the integration of the views of service users into models of care.^45^

## Discussion

### Main findings

This systematic review identified 52 studies providing information on the palliative care needs of adults with intellectual disabilities and the barriers and facilitators this population face in accessing palliative care. All of these studies were conducted in high-income countries, the majority in the United Kingdom. Qualitative methods were most commonly used. The studies mainly reflected experiences from community or residential settings, and there was a strong bias towards the experiences of health care professionals. From a total of 2970 participants, just 31 people with intellectual disabilities were included. Identified needs included physical needs, psychosocial and spiritual needs, and information and communication needs. Barriers and facilitators were associated with education, communication, collaboration, and health and social care delivery.

### What this study adds

This systematic review provides the first synthesis of the palliative care needs of adults with intellectual disabilities and the barriers and facilitators this population face in accessing palliative care. The current available evidence is almost exclusively reflective of the perspectives of health and social care staff, and the voices of people with intellectual disabilities and their families are lacking.

The palliative care needs identified in this review mirror those of the general population. The European Association for Palliative Care has also made this observation.^6^ However, meeting these needs is complicated by the challenges associated with this population. Understanding and recognition of these challenges for each individual, and anticipation of the problems they will face, are key to providing reasonable adjustments, a legal duty of all health and social care services^85^ that people with intellectual disabilities so deserve. Addressing education and communication barriers has potential to improve palliative care for this population. Closer attention is needed to how these can be addressed on a wider scale, with accompanying policies and guidelines to standardise practice. This needs to target staff at all levels, across both palliative care and intellectual disability services. Most studies in this review that evaluated interventions were small, and only provided information on the benefits to staff. Large-scale interventional studies exploring the effectiveness of interventions in improving palliative care for people with intellectual disabilities are needed. Building links between palliative care and intellectual disability services is crucial. Involving family members and carers is also important in delivering individualised care, as this provides an advocate that knows the person well and can facilitate communication with health care professionals. However, they must act in collaboration with the individual with intellectual disability, involving them in decision-making as much as possible, and facilitating their right to autonomy.

The WHO acknowledges the need to improve access to end-of-life care for hard to reach groups.^86^ Indeed, it has been stated that ‘how we care for the dying is an indicator of how we care for all sick and vulnerable people’.^87^ Many of the identified barriers and facilitators to accessing palliative care are likely to be encountered by other socially disadvantaged groups.^88,89^ The conceptual model presented in this article may therefore be of use to service developers and policy makers in other areas. Thus, policy makers have much to learn from acknowledging the barriers faced by this population.^79^

### Limitations

Only five electronic databases were searched. The hand search included only two intellectual disability specific journals and search of the grey literature was limited to two online databases. However, no date or geographical limitations were applied to the database search, which widened its scope. Although multiple researchers were involved in the screening process, E.A. conducted data extraction and grading of quality independently, presenting a source of bias. Yet, the use of a predefined data extraction template and a structured critical appraisal tool acted to reduce subjectivity as much as possible. It was a challenge judging the quality of such a heterogenous group of studies with one tool; however, Hawker et al.’s checklist^34^ is appropriate to appraise multiple study designs. Loosely defined populations meant in some cases it was difficult to separate participants that were eligible for inclusion alongside those that were not. As far as possible, data were only cited where the source of the information was clear, and Figure 2 only includes participants that were clearly defined. The narrative approach used to synthesise the evidence may be seen as subjective and therefore open to bias.^36,37^ However, the use of NVivo enabled a more systematic approach to this. The data predominantly reflect the experiences of health care professionals in high-income countries so may not be generalisable to other settings.

### Recommendations for future research

There is a need for high-quality studies that not only describe the problems faced by people with intellectual disabilities near the end of life, but evaluate the benefits of specific interventions. The paucity of research in this area further disenfranchises an already marginalised group. Areas to focus on are education, communication and service development. Potential initiatives that could be evaluated in future studies include the effectiveness of toolkits, link nurses and training days on patient care. Other approaches could be developing assessment tools such as the DisDAT and PALLI. However, this review demonstrates a lack of suitable and validated outcome measures for people with intellectual disabilities and palliative care needs. It is vital that these are developed in order to prove the effectiveness of proposed initiatives in improving care for this population.

Future research must involve people with intellectual disabilities and their family members as active participants. This will provide a deeper understanding of the inequalities experienced by this population and of their priorities and perspectives of what ‘good palliative care’ looks like. Research should also be encouraged on an international scale and involve low- and middle-income countries. This would help in getting palliative care for people with intellectual disabilities on the global health agenda.

## Conclusion

Addressing the inequalities in accessing palliative care for people with intellectual disabilities should be an urgent priority, particularly given the ageing population and concomitant co-morbidities. Currently, much of the empirical research in this area has focused on describing the problem. However, there are examples of good practice or pioneering initiatives that have potential to address inequalities in accessing palliative care. Such initiatives need to be evaluated through high quality, appropriately funded research that involves people with intellectual disabilities and their carers, as well as relevant health care professionals. Reliance on ‘committed individuals’^78^ or a ‘holistic philosophical approach’^7^ is not sustainable. Reasonable adjustments should be standard practice and not award-winning exceptions.^78^ Good practice in palliative care for this population needs to be standardised and implemented in all health care settings, across specialties and into mainstream services.

## Supplemental Material

15-05-20_Revised_supplementary_data_-_appendices – Supplemental material for The palliative care needs of adults with intellectual disabilities and their access to palliative care services: A systematic review Click here for additional data file. Supplemental material, 15-05-20_Revised_supplementary_data_-_appendices for The palliative care needs of adults with intellectual disabilities and their access to palliative care services: A systematic review by Emily Adam, Katherine E Sleeman, Sarah Brearley, Katherine Hunt and Irene Tuffrey-Wijne in Palliative Medicine
