# Supplementary material for: The palliative care needs of adults with intellectual disabilities and their access to palliative care services: A systematic review
Source: Palliat Med. 2020 Jun 17;34(8):1006–18. doi: 10.1177/0269216320932774 (PMC7596767; doi:10.1177/0269216320932774)
Supplement: 15-05-20_Revised_supplementary_data_-_appendices – Supplemental material for The palliative care needs of adults with intellectual disabilities and their access to palliative care services: A systematic review [file 15-05-20_Revised_supplementary_data_-_appendices.pdf]

Supplementary data: appendices

**Appendix 1**	Example of search strategy: MEDLINE
**Appendix 2**	Data extraction table
**Appendix 3**	Hawker et al's critical appraisal tool
**Appendix 4**	Grading of quality

Appendix 1 : Example of search strategy: MEDLINE

Database: Ovid MEDLINE(R) and Epub Ahead of Print, In-Process & Other Non-Indexed Citations and Daily <1946 to June 11, 2019>
Search Strategy:

Palliative care:

1 exp Terminal Care/ or caregiver$.mp. or bereave$.mp. or inpatient.tw. or attitude to death.mp. or end of life.af. or hospice$.mp. or terminally ill.mp. or palliative$.mp. or Advance Care.af. or palliat.af. or advanced.af. or (morphine and cancer).af. or cancer pain.af. [mp=title, abstract, original title, name of substance word, subject heading word, floating sub-heading word, keyword heading word, organism supplementary concept word, protocol supplementary concept word, rare disease supplementary concept word, unique identifier, synonyms] (841709)

Intellectual disability:

2 exp Intellectual Disability/ (92889)
3 exp Learning Disorders/ (21509)
4 (mental* or developmental* or intellectual* or cognitive or learning).ab,ti. (1093120)
5 (retard* or disab* or deficien* or delay*).ab,ti. (1185948)
6 4 and 5 (153599)
7 2 or 3 or 6 (227297)

Needs:

8 exp "Health Services Needs and Demand"/ (57615)
9 exp Needs Assessment/ (28555)
10 (needs assessment or assessment of healthcare needs or assessment of health care needs or unmet needs).ab,ti. (10359)

Access:

11 exp Health Services Accessibility/ (104713)
12 exp Healthcare Disparities/ (14484)
13 (access or access to care or barriers to care or obstacles or equity of care or inequity or inequality or allocation of health care resources or rationing or gate keeping or evaluation of care or quality of health care or assessment of need or unmet need or health care need or pattern of referral or referral).ab,ti. (420591)

Needs OR Access:

14 8 or 9 or 10 or 11 or 12 or 13 (570630)

Palliative care AND Intellectual disability AND [Needs OR Access]:

15 1 and 7 and 14 (894)

****

Appendix 2: Data extraction table

Author/date	Title	Published in	Country	Study design	Aim	Population	Setting	Needs	Facilitators	Barriers
(Bailey et al., 2016)	Surveying community nursing support for persons with an intellectual disability and palliative care needs	British Journal of Learning Disabilities	Ireland	Descriptive cross sectional survey	To describe the provision of community nursing support for persons with an ID and palliative/end-of-life care needs.	Public Health Nurses (PHN), Community Nurses, Practice Nurses, Hospice at Home Nurses and Palliative Care Nurses (n=33)	A health service region in Ireland	Information, advice, emotional support, symptom management, nursing care at home, nutrition management, pressure area care, referral to agency, hospice referral.	Family involvement/ support, nursing experience of ID, teamwork/ collaboration between care providers, support from ID services, personal attributes of professionals: compassion, communication, knowledge, honesty. Building a relationship with patient/ family.	Lack of cooperation from family, lack of experience/ education/ knowledge/ communication skills, lack of co-ordination within/ between services, delay in recognising end of life, delay in referral to PC, lack of ACP, poor referral information, lack of knowledge about patient.
(Bekkema et al., 2014a)	Respecting autonomy in the end-of-life care of people with intellectual disabilities: a qualitative multiple-case study	Journal of Intellectual Disability Research	Netherlands	Qualitative interviews	To describe how caregivers and relatives shape respect for autonomy in the end-of-life care for people with ID.	16 relatives & 31 HCP involved in the care of 12 recently deceased patients with ID	10 ID care provider organisations in different parts of the Netherlands.	Information, autonomy, need to communicate wishes, involvement in decision making, physical care.	Knowledge/skills in end of life care: communication skills, recognising EOL care needs, ability to interpret symptoms. Education.	Lack of HCP experience, knowledge and skills.
(Bekkema et al., 2014b)	Training needs of nurses and social workers in the end-of-life care for people with intellectual disabilities: A national survey	Nurse Education Today	Netherlands	Postal survey	To gain insight into the quality of end-of-life care, past vocational training, training needs and expert consultation opportunities of nurses and social workers working in ID care services.	Nursing staff (n=93), Social workers (n=37).	Working in care services for people with ID in the Netherlands.		Collaborative working between ID and SPC services.	Lack of training/education in EOL care. Lack of access to external experts i.e. SPC.
(Botsford, 2004)	Status of End of Life Care in Organizations Providing Services for Older People with a Developmental Disability	American Journal on Mental Retardation	USA	Survey (paper & electronic questionnaire)	To establish a baseline of information on the current status of end of life care in organizations serving older people with an ID and their families.	160 professionals providing services to older people with an intellectual disability and their families.	National organizations delivering a range of services to people with ID		Training staff, coordinated system of care in the community, availability of resources on EOL care for HCPs/ family/ carers/ patients, policies & guidelines, ACP	Availability of direct care staff, Availability of nursing/ medical staff/ funding, Staff untrained in EOL care, Anxiety about responding to families, Concerns about liability.
(Brown et al., 2003)	'Please don't let it Happen on my Shift!' Supporting Staff who are Caring for People with Learning Disabilities who are Dying	Tizard Learning Disability Review	UK	Qualitative: case series, interviews, observation of team meetings.	Not stated	Case series: 21 people with ID and terminal illness who had died. Interviews: 31 care staff, 16 service managers, 18 health care/ID professionals, 3 service users, 6 family members, 8 local community representatives, 1 external representative. Meetings: 5 staff meetings, 1 group of older people with ID.	12 services that have cared for a person with ID during a terminal illness. Staffed housing (3), residential home (3), hospital (2), community (2), hospice (1), respite service (1).	Pain, care at home, information	Speech and language therapist to aid communication, relationships/knowing the patient,	Diagnostic overshadowing, late diagnosis, difficulty communicating, reluctance to investigate, uncertain trajectory, lack of co-ordination between health & social services, absence of formal protocols, lack of clear legislation (e.g. consent, mental capacity, proxy decision makers), fear of disclosure/ collusion, conflict between carers and family members

(Cartlidge and Read, 2010)	Exploring the needs of hospice staff supporting people with an intellectual disability: a UK perspective	International Journal of Palliative Nursing	England	Qualitative: 2 questionnaires, 1 focus group discussion	To identify what educational and preparation skills hospice staff felt were needed to enable them to feel confident in providing specialist palliative care to people with ID.	Questionnaire: inpatient unit staff (n=26). Focus group discussion: inpatient unit & H@H staff (n=17)	North Staffordshire hospice: 28 beds, day hospice, H@H & education unit.	Management of multi-morbidities	Access to professional expertise, collaborative working alongside ID nurses, ID link nurse, access to resources, ID toolkit, collaborative working with family, staff support	Communication challenges, lack of experience/ knowledge/ skills/ education/ training, difficulty determining patient understanding, collusion, difficult family dynamics, challenging behaviours of patient, uncertainties in managing co-morbidities, stigma.
(Codling et al., 2014)	End-of-life training for paid carers working with people with learning disabilities	British Journal of Community Nursing	England	Focus group discussion & evaluation forms	To implement and evaluate a study day for paid carers on end-of-life care for people with ID.	Paid carers working with individuals with ID (n=43)	Residential care homes, day centres, respite care centres, supported living settings. East & West Berkshire.		Person-centred care/end-of-life plans, Supervision, Assessment tools, Training/guidance about services available and how to access, emotional support for staff	Views of people with learning disability differ from parents, Opportunities to talk about death, Recognising and treating pain, too many professionals /do not understand the roles. Inadequate training heightening communication fears.
(Cross et al., 2012)	Practical Approaches toward Improving End-of-Life Care for People with Intellectual Disabilities: Effectiveness and Sustainability	Journal of Palliative Medicine	England	Staff training sessions. Evaluation: discussions with steering group, face-to-face and telephone interviews.	To capture the learning from a 3 year project aimed at exploring ways of increasing access to palliative care services by people with ID within one geographical area.	ID and palliative care staff	South West London: 4 hospices & 228 care homes		Collaboration between ID and SPC services, PC training for ID staff.	Lack of understanding of each other's role between palliative care professionals and ID staff, tension between specialism and generic working across boundaries, lack of inclusion of people with ID into mainstream services - a view that they require specialist services.
(Dunkley and Sales, 2014)	The challenges of providing palliative care for people with intellectual disabilities: a literature review	International Journal of Palliative Nursing	UK	Literature review	To explore the challenges of providing effective palliative care for people with ID.	Adults with ID & palliative care needs and those caring for them	90 publications from North America, Europe, and the UK.	Communication needs. Time: everything takes longer. Expression of views/ right to information/ involvement in one's own care, having friends and family around, being occupied, physical comfort.	Collaboration between ID and SPC services, early referral of people with ID to SPC, emotional support for staff, training (=confidence/ better communication), knowing the patient, use of assessment tools	Lack of staff confidence and knowledge. Communication: assessment, consent, patient comprehension, collusion, involvement of patients in their end-of-life care/ ACP. Relatives as gatekeepers/ often want to protect. Paternalistic approach of health care. Diagnostic overshadowing.
(Flynn et al., 2015)	Psychosocial experiences of chronic illness in individuals with an intellectual disability: A systematic review of the literature	Journal of Intellectual Disabilities	UK	Systematic review	To identify, evaluate and synthesize literature exploring the psychosocial experiences of chronic illness in adults with ID.	2 pts with ID and life-limiting illness: 30 F with CP &NHL, 44M with CP & advanced penile cancer.	UK, setting not stated.	Spiritual, psychological, physiotherapy, physical effects of surgery-loss of mobility, support from family/friends.		Delayed diagnosis, reluctance to investigate, use of complex language by health care professionals, placed in wards or homes for older people.
(Forrester-Jones et al., 2017)	People with intellectual disabilities at the end of their lives: The case for specialist care?	Journal of Applied Research in Intellectual Disabilities	England	Mixed methods: observations, interviews, questionnaires, costs analysis	To explore the organizational context of a specialist palliative and end-of-life care home to see how it related to the quality of life of people with ID who were approaching the end of their lives.	Residents (n=9), staff members (n=15)	A specialist, high dependency registered nursing home for older people with ID, South England.		Holistic approach: small number of residents allowing more individual staff–client time, provision of activities & therapies in addition to clinical & social care. Person-centred approach: incorporating dignity, knowing people well, compassion/ empathy, especially when communication difficult. Staff making efforts to offer choice & independence. Homely social environment. Emphasis on early admission following terminal diagnosis to avoid transition close to death, recognition of 'unknown time-factor,' long term care until you die - unlike hospice. Supportive	People with ID being "misplaced in older people's homes living alongside much older and more incapacitated people."

									management: responsive to staff needs, willingness to organize training, familiarity with residents. Willingness of staff to go “extra mile” to improve residents’ quality of the life. Adequate funding: this service costs!	
(Friedman et al., 2012)	Unique and Universal Barriers: Hospice Care for Aging Adults with Intellectual Disability	American Journal on Intellectual and Developmental Disabilities	USA	Literature review	To better understand the unique barriers to end-of-life care for aging adults with ID.	Adults with ID/end of life care needs and those caring for them	42 articles (US, UK, Belgium, Ireland, Netherlands, New Zealand).	Information needs - considers cultural and communication differences.	Training of care providers	Delay in diagnosis, Late referral to hospice, Reluctance to broach subject, financial constraints, lack of resources, lack of knowledge, carer familiarity with PC, Incomplete communication of treatment options, Conflicts between carers and surrogate decision makers, Ethical dilemmas regarding over/ undertreatment, Challenges with informed consent.
(Gilbert et al., 2007)	Supporting people with intellectual disability in the cancer journey: The ‘Living with cancer’ communication pack	European Journal of Oncology Nursing	UK	Evaluation: document review, focus groups, postal questionnaire, telephone interview.	An evaluation of the effectiveness of the ‘Living with cancer' communication tool.	Focus groups: 6 adults with ID. Questionnaires: 18 ID professionals. Telephone interviews: 7 ID professionals.	ID services across UK.		Use of a tool designed by experts, encompassing ‘total communication techniques’ to aid communication and discussion of sensitive issues.	
(Grindrod and Rumbold, 2017)	Providing end-of-life care in disability community living services: An organizational capacity building model using a public health approach	Journal of Applied Research in Intellectual Disabilities	Australia	Mixed methods: online survey, focus groups, interviews.	To identify structural, cultural and practical factors that influence end-of-life outcomes for people with ID in the community.	ID professionals in the community: survey (n=179), focus groups (n=25), interviews (n=7)	Community ID services, Victoria, Australia	Asking, recording and respecting preferred place of care and death.	Past experience. Leadership skills. Staff support - emotional & educational. Teamwork/ support from colleagues. Committed staff working additional hours. Clear policies/ protocols. Relationship with patient. Staff resilience. Public health approach.	Staff attitudes: dying best managed by medically trained, not in my job role, death should be in hospital or nursing home. Lack of awareness of policy/ protocol. Lack of training/ experience. Inadequate staffing levels. Patients not offered choice. Absence of coordination between ID staff & SPC. Juggling needs of multiple stakeholders. Staff stress/ feeling unsupported. Fear of impact on other residents: grief/ resources taken up by dying. Absence of confident formal leadership. Inconsistent practice/ policy between settings.
(Hunt et al., 2019)	End-of-life care in intellectual disability: a retrospective cross-sectional study	BMJ Supportive & Palliative Care	UK	Retrospective cross-sectional survey	To describe the end-of-life care outcomes for adults with ID living in residential care in the UK using the VOICES-SF questionnaire and compare these with the general population	157 care staff involved in care of person with ID in last months of life	38 providers of residential care for adults with ID across the four countries of the UK.	Pain	GP and community nurse input. Recording PPD: Of those that did, the majority died in what was reported to be the preferred place.	Patient lack of awareness that they were dying. Preferred place of death not recorded. Lack of involvement in their own care. Hospital based care inferior to community-based care with lower levels of support from external services.

(Kim and Gray, 2018)	Palliative Care Experiences and Needs of Direct Care Workers	Journal of Palliative Medicine	USA	Focus groups	To explore direct care workers experience with PC and their perceived challenges.	Direct care workers serving people with ID (n=54)	5 non-profit community-based organisations providing residential and day services for people with ID in urban and suburban areas within or surrounding a large Midwestern city.		Empathy. Extra attention. Adaptation in care/ taking more time. Peer support/ teamwork.	Difficulties in communication with patient. Anxiety in anticipating death. Limited organisational resources or support; understaffing, inadequate communication between staff of different grades/ specialities, insufficient training.
(Kinley et al., 2018)	Development and implementation of the Steps to Success Palliative Care programme in residential care homes for people with a learning disability in England	International Journal of Palliative Nursing	England	Literature review, meetings with ID, SPC & care home staff.	To develop and implement a palliative care programme that would meet the needs of people with a LD, their families and care home staff.	Care home, ID and SPC staff.	30 care homes		Delivery of an evidenced based formal staff education programme: improved identification/ assessment/ symptom management/ ACP/ changed attitudes towards EOL. Collaboration between services. Learning at an individual, organisational and systems level. Ongoing facilitation/ training new staff to achieve sustainability.	
(Lindop and Read, 2000)	District nurses' needs: palliative care for people with learning disabilities	International Journal of Palliative Nursing	England	Focus groups, questionnaires	Identify national PC issues, and current PC services provided by DNs for people with ID in North Staffordshire, UK.	Focus groups:12 DNs undertaking a diploma in PC. Questionnaires: 106 DNs (54% had contact with ID patients with PC needs).	North Staffordshire, DNs in community	Pain, information, care in the community.	Education and training of district nurses.	Communication difficulties. Lack of experience/ training.
(Marriott et al., 2013)	Good practice in helping clients cope with terminal illness	Learning Disability Practice	England	Case study	To provide an example of good practice in end of life planning and palliative care for a person with ID.	Male in 50s with lung cancer and mild ID	England: hospital & community.	Dietary & nutrition needs. Assistance with complicated medication regime. Pain. Wish to die at home.	Thorough and timely investigation. Allowing enough time for appointments to ensure understanding. Translating medical terminology into simpler concepts to maximise involvement in decisions. Continuity of care with same hospital consultant. Allocation of CLDT nurse. Collaboration between services. Family involvement. Building relationships with patient. Anticipating future needs/ACP, documenting wishes. Regular GP & DN visits. GP as key player.	
(McKechnie, 2006)	What does the literature tell us about death, dying and palliative care for people with intellectual disabilities?	Progress in Palliative Care	New Zealand	Literature review	Not stated.	Not stated.	Not stated.			Difficulties in detecting disease in people who are non-verbal, diagnostic overshadowing, delayed diagnosis. Lack of involvement of the individual in their care: decisions being made for them by family members or health practitioners. Inadequate education of formal and informal carers. Changing healthcare needs/ disease profiles as people with ID are living longer. Health professionals making assumptions about understanding/ ability. Legal barriers around informed consent, capacity and competence.

(McLaughlin et al., 2014)	Developing a best practice model for partnership practice between specialist palliative care and intellectual disability services: A mixed methods study	Palliative Medicine	UK	Questionnaires & interviews	To develop a best practice model which would guide and promote partnership practice between specialist palliative care and ID services	Questionnaires: ID services (n=10), SPC services (n=22), ID nursing homes (n=15). Interviews: 30 HCPs who had provided EOL care to adults with ID.	Primary and secondary care in one region of the UK.		Partnership working between ID and SPC: easy to contact, regional meetings, joint study days, joint working/shadowing in both clinical areas. Co-ordination and continuity of care, familiarity with the patient. Educating, supporting and empowering staff. The role of the GP: early identification of need and specialist referral.	'Medical' versus 'social' model of care, time constraints, limited/ late referrals to SPC, mistrust/ conflict between services.
(McNamara et al., 2019)	Creating person-centred support for people with intellectual disabilities at the end of life: An Australian qualitative study of unmet needs and strategies	Journal of Intellectual Disabilities	Australia	Interviews	Use the perspectives of health professionals and paid carers to document the range of needs adults with an ID experience during the last months of their lives	Nurses (9), social workers (4), occupational therapists (3), disability residential accommodation managers (6), disability support workers (2), psychologists (2)	Community PC organization, community nursing organization, disability accommodation services, public hospital, country health service. Western Australia.	ACP. Enabled to die in familiar surroundings: dying at home. Full time (24/7) care as illness progresses. Pain. Symptom control. Physio & OT needs: repositioning/ wheelchairs. Person-centred care: awareness of social/ethnic background, personal history and modes of communication. Opportunities for choice. Religious/ spiritual needs. Nutrition.	Open communication with the person and their family. Good staff support and bereavement care. Familiarity with patient. Collaboration between MDT. Continuity of HCP. Greater openness/ awareness & strong advocacy. Identify case coordinator/manager, shared care between services, training ID staff in PC/training PC staff in ID, available PC guides/policies, routine use of disability-oriented pain/symptom assessment tools, early identification & referral to PC. Use speech therapists to assist with interpretation. Communication aids and electronic devices. Accompanied by staff/family during hospital stays. Reduce transitions and create familiar environments. GP as key player.	Paternalistically protecting other residents from grief. Poorly trained staff. High staff turnover. Communication issue. Diagnostic overshadowing. Lack of communication between hospital & community services. Lack of ACP = unnecessary ED admissions. Lack of funding. People with ID 'hidden' by society.
(Moro et al., 2017)	Agency, social and healthcare supports for adults with intellectual disability at the end of life in out-of-home, non-institutional community residences in Western nations: A literature review	Journal of Applied Research in Intellectual Disabilities	USA	Literature review	To summarize the literature on the agency, social and healthcare supports that impact the care of adults with ID at the end of life	Adults with ID/end of life care needs and those caring for them	All health & social care settings	Transitioning needs: home/ residential care/ nursing home/ hospital. Expressing & documenting wishes. Care at home. Information. Help to understand diagnosis & prognosis. Proximity of family accommodated.	Time to build relationships between staff, patient and family. Partnerships between ID and PC. Education.	Lack of staff training. Collusion. Proxy decision making. Reluctance to investigate/ late diagnosis. Under AND over treatment. Transportation barriers and a lack of accessible medical equipment in hospitals: hindering admission for medical care. Poor communication/ negative rapport with patient/ carers.
(Morton-Nance and Schafer, 2012)	End of life care for people with a learning disability	Nursing Standard	England	Face to face interviews	To explore the experiences of LD and district nurses caring for people with a LD at the end of their lives	Community learning disability nurses (n=3), district nurses (n=3), all with experience of providing care for people with ID at EOL.	Community	Being empowered to make choices. Effective and dignified management of symptoms. Physical comfort. Physical and mental pain. Time.	Collaborative working between teams/services. Person-centredness, planning, preparation, outreaching and sharing of information with other HCPs. Training in EOL care and policy. Emotional support for staff and residents.	Staff attitudes: unwillingness to put in extra time, complacency, divide between services, protective/ paternal leading to withholding of info. Specific choices/ decisions not documented creating conflict/ preventing collaborative working. Patient non-compliance with medication. Diagnostic overshadowing. Being reactive rather than proactive. Fragmented communication between HCPs/ patient/ carers, limited sharing of info. Patients' inability to communicate their needs. Inexperience, lack of understanding/ skills/ training. Discomfort when

										discussing death and dying directly with the patient.
(Ng and Li, 2003)	A survey exploring the educational needs of care practitioners in learning disability (LD) settings in relation to death, dying and people with learning disabilities	European Journal of Cancer Care	England	Questionnaires	To explore whether care practitioners have the knowledge and skills to care adequately for dying people with LD in residential settings	25 qualified care practitioners working in residential settings	Residential care homes, 2 NHS Trusts in England.	Psychosocial care, pain and symptom control, use of drugs. Wound care. Cultural awareness. Care after death. Support for family.	Verbal and non-verbal communication skills. Education in EOLC skills. Updating nursing curriculum.	Lack of relevant education and training. Staff understanding of diagnosis and causation of death, particularly non-malignant diseases. Inability to recognise dying. Social care vs medical care - are carers too familiar with social side and lacking clinical skills?
(Northway et al., 2019)	Nursing care at end of life: a UK-based survey of the deaths of people living in care settings for people with intellectual disability	Journal of Research in Nursing	UK	Questionnaire	'What are the levels of support provided by nurses in the last months of life for people with ID?'	38 ID care providers in the UK	Social care residential setting or nursing home for people with ID		Training and education targeted towards community and hospital-based nursing staff: they were shown to have significant involvement in the care of people with ID in last months of life.	
(O'Regan and Drummond, 2008)	Cancer information needs of people withintellectual disability: A review of the literature	European Journal of Oncology Nursing	Ireland	Literature review	A review of the literature with a specific focus on the cancer information needs of people with an ID	Adults with ID and cancer, those caring for them.	All health & social care settings	Cancer information materials that can be accessed and understood by people with an ID. Psychological distress when receiving bad news. Autonomy. Management of side effects: nausea, vomiting, fatigue and pain. Anxiety.	Communication skills/BBN training for ID staff not just oncology. Involvement of family members/carers to aid communication, informed decision making and autonomy. Collaboration/ interdisciplinary approach between oncology, community teams, PC teams and ID services. ID organisations providing access to specialist information and cancer services. Visits to radiotherapy and chemotherapy units prior to treatment. Information leaflets with a wide range of visual content. Education in use of augmentative and alternative communication systems: sign language, picture symbols, eye pointing to support communication and information needs. Hospital policies and protocols.	Poor communication skills. Social taboos/ assumption they will not comprehend resulting in withholding information regarding diagnosis and prognosis.
(O'Sullivan and Harding, 2017)	Transition: the experiences of support workers caring for people with learning disabilities towards the end of life	BMJ Supportive & Palliative Care	England	Focus group	To provide a better understanding of the experience of support workers, as paid carers, caring for adults with LD nearing the end of life in residential settings.	13 residential support workers with direct experience of caring for a person with ID towards the end of life	3 independent service provider settings for people with ID in London		Collaboration with other services. Partnership between ID and SPC services. Support from management within own organisation. Adequate staffing at night. Collaboration/ relationship with family. Early involvement of SPC. Education and training. Emotional support for staff: bereavement.	Denial and a lack of preparedness for seeing residents entering the dying phase of their lives. Lack of access to extra training and support. Standard practice for support workers to be on their own with residents when they were dying. Lack of organisational policy/ guidelines.

(Read and Cartlidge, 2012)	Critical reflections on end-of-life care for people with intellectual disabilities: a single case study approach.	International Journal of Palliative Nursing	England	Case study	To explore the challenges to providing effective end-of-life care for a person with an ID by using critical reflection from a nursing care perspective on an illustrative case study.	36 F with ID and brain tumour	Hospice, England	Day hospice: social contact with others. IPU: symptom assessment or management, respite. Spiritual needs.	Project worker acted as conduit between generic end-of-life care and ID services. Fulfilment of any last wishes to augment a more peaceful death. MDT approach, best interest meeting.	
(Reddall, 2010)	A palliative care resource for professional carers of people with learning disabilities	European Journal of Cancer Care	England	Case study	Use of a case study to help illustrate the value of compiling a resource booklet for professional carers of people with LD.	72 M with ID & prostate Ca	Community, England	Regular contact with GP, medication needs. Loss of appetite. Declining mobility. Urinary incontinence. Pain. Oral thrush. Fear of strangers. A desire to stay at home.	Provision of easy to understand & easily accessible booklet on palliative care for staff in a learning disability care home. Presence of key worker that knows patient for consultations & scans. Interdisciplinary working, shared expertise of ID and SPC. Community team involvement: DNs & Macmillan nurse. MDT reflective session after death.	Carers working in isolation, without access to information/ guidance. Care home manager unaware of how to access SPC team. Poor record keeping. Poor referral info.
(Ronneberg et al., 2015)	Promoting Collaboration Between Hospice and Palliative Care Providers and Adult Day Services for Individuals with Intellectual and Developmental Disabilities	Journal of Death and Dying	USA	Online surveys & focus groups	Not stated.	Surveys: ID service providers (n=103), hospice/PC providers (n=87). Focus groups: adult day service providers (n=10), hospice/PC providers (n=7).	Community, residential home & hospice, USA		Education around recognition of nonverbal symptoms of pain. Well-coordinated services, knowledgeable about one another	Lack of established relationships between ID and hospice services. Few clients receiving dual (hospice and ID) services. Need for training and education for both hospice and ID services. Lack of clearly established and communicated DNR policies, lack of support/ guidance from state government around DNR policies. Misconception that PC patients must always be transferred to hospice for care. Hospice providers limited experience in working with persons with ID. Confusion around funding and regulations. Difficulty having client with extensive medical needs. Staff/family attitudes about death and dying/resistance to hospice philosophy.
(Ryan et al., 2016)	The nature and importance of quality of therapeutic relationships in the delivery of palliative care to people with intellectual disabilities	BMJ Supportive & Palliative Care	Ireland	Focus groups	To describe palliative care delivery to people with ID in a region in Ireland and to assess the population's palliative care needs	91 staff: PC services (n=27), ID services (n=64)	One Health Service Executive area in Ireland	Familiarity, time allowed to build relationships with new HCP. Pain.	Importance of patient-professional relationship: building a therapeutic relationship. Continuity of relationship, knowing the individual, building trust. ID staff familiarity with patient: getting to know them over time. Strengthening interface between PC & ID services.	PC staff unfamiliarity with patients with ID. Over-reliance on carers for communication/ information/ 3 way relationship: prevented PC staff building proper therapeutic relationship. PC staff failure to form authentic relationships with people with ID = poorer quality of care, harder to overcome collusion from family.

(Ryan and McQuillan, 2005)	Palliative care for disadvantaged groups: people with intellectual disabilities	Progress in Palliative Care	Ireland	Literature review	A review of the palliative care needs of people with ID	People with ID and PC needs, those caring for them.	All health & social care settings	Pain. Time to express oneself. Emotional distress. Symptoms expressed as objectively observable signs, rather than spontaneous complaints: e.g. chest pain, dyspnoea, dyspepsia, micturition, hearing and visual loss expressed as irritability, inactivity, loss of appetite and sleep disturbance. Intestinal obstruction in bowel cancer: often undetected due to lack of expressed symptoms & visual signs. Right to information/autonomy. Provision of care on place of choice.	Body map and photographs to communicate symptoms. Use of creative media, art, music, life-story work & audiotapes to overcome communication challenges. Adaptation of standard communication models e.g. BBN where subtlety of warning shot will not be detected. Involvement of carer who knows the individual. Symptom assessment tools for people with ID. High index of suspicion for complications in certain conditions e.g. bowel cancer & obstruction. Effective liaison between ID & PC services. Emotional support for ID staff.	ID patients accessing PC not offered full range of services: e.g. complementary therapy and hospice day-care rarely offered. Cancer better supported than dementia = impact on those with DS. Withholding information, 'a conspiracy of silence.' Varying ability to understand the concept of death: difficult to assess cognitive level & communicate accordingly.
(Sampson et al., 2015)	European palliative care guidelines: how well do they meet the needs of people with impaired cognition?	BMJ Supportive & Palliative Care	Europe	Questionnaires	To review whether national palliative care guidelines in Europe consider pain assessment and management for the increasing numbers of adults with ID	Representatives of the European Pain and Impaired Cognition (PAIC) network	11 European countries			Palliative care guidelines do not universally consider the needs of people with ID. Lack of validated tools or those designed specifically for people with ID.
(Stein, 2008)	Providing Palliative Care to People with Intellectual Disabilities: Services, Staff Knowledge, and Challenges	Journal of Palliative Medicine	USA	Survey	To document the degree to which hospice and palliative care services were provided to New Jersey residents with ID, and the challenges in providing this care	Hospice and PC providers: hospice (n=19), hospitals (n=3). Residential care providers (n=55)	Community, hospice, hospital, New Jersey	Pain. Cognitive and motor deficits. "Human contact is critical." Encouraging autonomy to make decisions.	Strategies and tools for assessing capacity. Staff training. Linking of PC & ID services: educating each other/outreach. Consistent policies & standards.	Lack of staff knowledge and training (within both ID and PC services). Lack of experience with population. Lack of understanding of PC. Communication challenges with patient. Demanding family members. Additional costs (health insurance, medical equipment). Additional staffing. Lack of in-home caregivers. Fear of impact on other residents. Lack of staff support. Caregivers increasingly elderly = lack of able caregiver. Coordinating hospice & ID services. Reluctance by staff/carers to consider DNAR orders. Individuals presumed to lack decision making capacity.
(Sue et al., 2019)	Palliative care for patients with communication and cognitive difficulties	Canadian Family Physician	Canada	Literature review	To explore recent best evidence to address PC needs of people with ID. To help increase the confidence of caregivers and clinicians in providing PC to adults with ID using an easy-to-follow framework.	People with ID and PC needs, those caring for them.	All health & social care settings	Physical pain. Emotional distress. Communication needs. Information needs. Grief, opportunity to express grief nonverbally.	Collaboration between ID & PC services. Early referral to PC allows for longitudinal experience with time to build trust, familiarity, links with services, ACP. Individualised symptom assessment. Involvement of closest caregiver. Versatile symptom assessment tools e.g. DDAT, Abbey. Careful examination of distress patterns, thorough physical examination, and judicious use of diagnostic testing. Communication aids: books, visual tools. Allowing extra time.	**Caregiver:** Lack of understanding or regarding focus of care in advanced life-limiting illness. Ill-equipped for open communication regarding dying and death. Fear of upsetting patient. **HCP:** Communication barriers, over-reliance on carers for communication, assumption of lack of capacity, stress related to lack of experience. Inadequate use of standardised assessment tools. Difficulty distinguishing between physical pain & emotional distress = inappropriate use of analgesics. Overemphasis on role of cognitive ability in QOL = limitation of care options offered, i.e. undervaluing a

										patient's life. Unclear trajectories, unexpected recovery after deterioration = difficult to prognosticate. **Patient:** Difficulty understanding abstract concepts relating to death.
(Tuffrey-Wijne, 2003)	The palliative care needs of people with intellectual disabilities: a literature review	Palliative Medicine	England	Literature review	To answer the question: What are the palliative care needs of people with ID?	People with ID and PC needs, those caring for them.	All health & social care settings	Increase in age-related morbidities. Need for care in the community. Pain. Need to explain likely symptomatic course of an illness.	ID staff supported by primary care staff and palliative care specialists. Involvement of those who know patient well to help interpret individual pain behaviours. Symptom assessment tool for non-verbal communication of pain. Training and education for HCP and carers. Partnership between ID and PC services.	Diagnostic overshadowing. Difficulty obtaining consent, assuming patients lack ability to consent.
(Tuffrey-Wijne et al., 2007a)	End-of-Life and Palliative Care for People with Intellectual Disabilities Who have Cancer or Other Life Limiting Illness: A Review of the Literature and Available Resources	Journal of Applied Research in Intellectual Disabilities	UK & Netherlands	Literature review	To review the literature around the need of people with ID for palliative care	Adults with ID & PC needs	All health & social care settings	Right to know diagnosis and prognosis. Information in accessible format. Pain. Need for social network: friends & family. Familiar environment with familiar people.	Committed and well supported staff. Collaboration between HCPs, ID staff & family. Openness and disclosure. Use of tools/pictures to assess pain. "Skilled observation together with an intimate knowledge of the person." ACP: advance directives. Careful consideration of capacity involving MDT, carers & family. Equipping ID residential services with resources & training to provide PC. Familiar environment with familiar people. Training manuals, protocols, guidelines.	Late presentation, diagnostic overshadowing, difficulty identifying symptoms, resulting in advanced disease with severe symptomatology. Lack of ID staff experience/training in caring for dying. Communication difficulties hindering recognition of pain: overreliance on carers. Overtreatment: reluctance to withdraw futile treatments for fear of litigation. Undertreatment: not giving option of life-prolonging treatment. Understaffing, lack of training, staff anxiety. Lack of evidence base.
(Tuffrey-Wijne et al., 2007b)	Palliative care provision for people with intellectual disabilities: interviews with specialist palliative care professionals in London	Palliative Medicine	England	Interviews	To identify the issues affecting the delivery of services by specialist palliative care professionals to people with ID in London	SPC professionals (n=32). (Only 24 had seen patients with ID within PC).	3 hospices & 3 hospital PC teams in Greater London, representing suburban as well as inner city communities	Personal care needs: washing etc. (independence inhibited by illness). Fear/anxiety, existential distress. Emotional support for family. Community care to avoid change of environment. Mental health problems.	Support for care staff: educational, building confidence. Liaising with ID professionals who know the patient well: particularly helpful with mental capacity and consent. Early referral to PC. Taking time to build relationship/trust = aids examination & assessment. Good background info on individual patient more valuable than formal training. Increase referrals: one learns best through experience. Collaboration between carers, PC & ID services: awareness of each other.	**Social:** Overprotectiveness from family. Poor communication with family/carers. Lack of NH that can meet needs of ID patients. **Emotional and cognitive:** Patient confusion/fear compounding communication barriers, inappropriate assessment of cognitive function. **Assessment:** diagnostic overshadowing, lack of information, examining, non-compliance with medication. **Impact on ward and staff:** grief & stress, need for extra time & staff. **PC staff:** lack of training/ experience, lack of knowledge of ID services. **ID staff**: patients referred late or not at all to PC.

(Tuffrey-Wijne et al., 2008)	Palliative care provision for people with intellectual disabilities: a questionnaire survey of specialist palliative care professionals	Palliative Medicine	England	Postal questionnaire	To gain insight into the current use of PC services by people with ID in London, to identify the prevalence and severity of problems faced by PC professionals, to identify strategies that will facilitate an improvement in access and delivery of PC services to people with ID.	SPC professionals providing PC services for adults (n=543). Of these, 67%, (n=364) had previously supported a patient with ID.	52 SPC services in London: hospice, community & hospital.	Symptom control, Terminal care, Support for carers/ family, Psychological support, Respite care, Day care. Fear. Pain. Emotional distress. Time. Familiarity.	Collaboration between services. Allocating link worker within each service. Formal training. Allowing time. Providing safe and predictable environment with familiar faces. Staff support. Family support.	Late diagnosis. Lack of referral to SPC. Fear of transfer to different setting if referred. Family protection/ fear of PC. Lack of awareness by both SPC and ID services of each other's existence and what each has to offer. Communication difficulties: understanding patient, symptom assessment, consent. Non-compliance with medication/care/ examination. Poor communication with family/carer. Patients' difficulty with comprehending their illness and treatment. Those that spend most time with patient had least training e.g. HCAs. Lack of background information. Difficulty finding suitable long term accommodation when unable to cope at home. Financial concerns.
(Tuffrey-Wijne and McEnhill, 2008)	Communication difficulties and intellectual disability in end-of-life care	International Journal of Palliative Nursing	England	Literature review drawing on 3 research studies conducted by the authors.	To map the communication difficulties experienced by people with ID within a palliative care setting.	People with intellectual disabilities, HCP & carers within a PC setting.	All health & social care settings	Information. Honesty. Time. Difficulty understanding abstract concepts.	Use of communication aids: Objects of reference, sign languages, symbol-based systems. Use of simple, non-euphemistic language, avoid jargon. Allowing extra time for consultations, addressing patient before carer.	**Communication:** Inability of patient to communicate verbally. Inability of HCP to confirm comprehension of diagnosis/treatment. Family collusion.
(Tuffrey-Wijne et al., 2009)	People with learning disabilities who have cancer: an ethnographic study	British Journal of General Practice	England	Ethnographic: participant observation	To explore the experiences and needs of people with LD who have cancer	Adults with ID and a cancer diagnosis (n=13)	Home, residential care home.	Lives defined by dependence on carers. Particularly vulnerable when ill. Anxiety precipitated by not understanding. Fear of cancer. Pain. Continuation of clear/ safe routines, treasured activities, relationships. Loneliness, isolation.	Preparation for appointments, family/ carer present. Explanation of procedures/ treatments in a way that is easy to understand. Familiarity with hospital/ clinic. Maintaining routine. HCP becoming integrated into safe/ trusted environment: a familiar face. Practical and emotional support for residential staff.	Diagnosis dependant on someone other than the patient noticing something is wrong or correctly interpreting behavioural changes: but often unnoticed or misinterpreted. Patient complaints ignored or disbelieved: attributing to 'attention seeking' behaviour. Medical staff guided by opinion of carers, without attempting to check views of patient. Not offered standard treatment options due to assumption that the person would not cope. People with ID unlikely to question treatment decisions. Information giving influenced by; personal opinion of the carer, desire to protect the person from distress and lack of skill and confidence in communicating with them. Family collusion. Acquiescence. HCP lack of experience: communication, fear of opioids, fear of death. Reluctance to ask busy doctors for more input.

(Tuffrey-Wijne, 2013)	A new model for breaking bad news to people with intellectual disabilities	Palliative Medicine	England	A 2-phase qualitative study: (1) focus group meetings, on-line focus groups, interviews; (2) structured feedback.	To develop a model for breaking bad news that meets the needs of people with ID	26 ID professionals & 34 general health professionals with experience of supporting an adult patient with IDs who had a life-limiting illness. (Also 21 people with IDs and 28 family carers but experience of life-limiting illness not stated in study).	Not stated.	People with IDs should be helped as much as possible to understand their situation. Helping someone with IDs understand and cope with bad news is a slow process with knowledge and understanding building gradually over time. Need time and support from the person's network: family & professionals. Emotional support.	Process of disclosing bad news should involve collaboration between those who understand the content of the information (health care professionals) and those who best understand the patient's communication needs (family). Use of a model for breaking bad news to people with ID. Emotional support for staff who may also be affected by bad news: in turn helps them to support the patient.	Family wish to protect the person with ID "I think I'd lie for his own good." Uncertainty about whether bad news should be given. Conflict between family and physician: "the family are not my patient." Conflict between family and ID nurse: barred from visiting. Difficult to assess how much someone can truly understand: PC consultant found they had not understood when it initially appeared that they had.
(Tuffrey-Wijne et al., 2016)	Defining consensus norms for palliative care of people with intellectual disabilities in Europe, using Delphi methods: A White Paper from the European Association of Palliative Care	Palliative Medicine	Europe	Delphi study using MM.	To define consensus norms for palliative care of people with ID in Europe	Professionals with expertise in PC, ID or both (n=80).	15 European countries, all health & social care settings.	Communication needs. Holistic approach: physical, emotional, social and spiritual needs. Counselling, support in maintaining social links with friends. Pain. Comorbidities. A right to life and a right to recognition of the value of their lives. Complicated grief.	ID services refer to PC when appropriate, PC services make necessary adjustments. HCP to recognise communication needs, strive to understand and to seek necessary training, support patient communication using methods that suit them, encouragement involvement in EOL decision making, ACP. Skills to recognise illness, deterioration and dying. Individual, person-centred care plans. Recognition that total needs exist as they do for patients without ID: holistic approach. Awareness that symptoms are expressed differently: collaboration with those who know the person well. Awareness of legal frameworks around capacity & consent. Involvement of family/carers/friends. Collaboration between services. Emotional support for family/carers. Education and training: for staff AND death education for patients. Prioritisation amongst policy makers and managers.	Diagnostic overshadowing.
(Tuffrey-Wijne et al., 2017)	Communicating about death and dying: Developing training for staff working in services for people with intellectual disabilities	Journal of Applied Research in Intellectual Disabilities	England	Stage 1: Semi-structured interviews. Stage 2: Development & evaluation of training course - feedback forms and questionnaires.	To assess staff training needs and to develop, trial and evaluate a training course on communicating about death and dying	Stage 1: 20 staff working in ID residential or supported living services (13 had supported people with ID who had died in the past 6 months). Stage 2: 114 interprofessional delegates, 108 completed feedback forms and questionnaires + 24 follow-up questionnaires at 3 months.	8 different ID residential or supported living services in London, UK		Organisational culture: junior staff felt more comfortable talking about loss, death and dying in settings where managers talked with staff and clients about death, providing positive role modelling. Staff training to improve the ability to talk about death and dying, not only with people with ID but also with each other. World Café approach: helping course delegates to talk about death and dying and "role-modelling" ways in which they could help their teams do the same. Involving people with ID by	UK ID services: workforce, especially junior care staff, is largely untrained - often learn on the job. Staff frightened and unprepared for dealing with issues of death and dying, avoidance of death-related conversations, concerned that communicating about death might cause distress, partly due to their own lack of skill. Not knowing how to break bad news. Avoidance of death-related communication had roots in organizational culture. Tendency among staff to "keep things positive." Lack of preparation and training for the job of care worker was striking. Lack of

									telling their own stories as part of staff training. Targeting training towards managers.	guidance for staff in death-related communication; staff communication was influenced by personal beliefs or opinions. Emotional support for staff to help them better cope with death and dying.
(Tuffrey-Wijne and Davidson, 2018)	Excellence in palliative and end-of-life care provision for people with intellectual disability	International Journal of Palliative Nursing	UK	Thematic content analysis	To identify the key characteristics of practice initiatives and case reports that have won a UK award for the outstanding provision of end-of-life care for people with ID (2008–2018).	People, projects or teams who were award-winners or highly commended (n=25).	Nursing/residential home, hospice, community, UK		**Individual and organisational commitment:** Enthusiastic individuals/leaders going beyond the call of duty. Adequate funding and policies. **Working together in collaboration:** between LD and PC, with patients, carers, families. Regular meetings. Mutual learning. Availability of required professionals, 24/7 care, continuity of care. **The person's story is at the heart of care:** Highly individualised care, creative & flexible, familiarity with patient, maintaining care in home environment, involving & supporting family. **Developing tools and staff training:** Inter-professional, regular, compulsory, conferences, use of GSF, tools and pathways, early referrals, ACP, adapting from mainstream.	Lack of national policies and adequate funding = postcode lottery, good initiatives not sustained when staff leave. "Good palliative care for people with ID should not be dependent on the fortunate presence of committed professionals with a passion for this area of care, or on being within the catchment area of one of these inspirational projects and services. Nor should it be up to a small charitable organisation like the PCPLD Network..."
(Vrijmoeth et al., 2016a)	Physician-Reported Symptoms and Interventions in People with Intellectual Disabilities Approaching End of Life	Journal of Palliative Medicine	Netherlands	Online retrospective survey	To determine the prevalence rates of physician-reported symptoms at the moment they recognise patient's death in the foreseeable future.	ID physicians who had provided PC to a deceased patient with ID (n=81).	All health care settings, Netherlands	Physical/symptom needs: fatigue, drowsiness, decreased appetite, shortness of breath, pain, anxiety, depressed mood, nausea, constipation, delirium. Medical needs: sedation, wound care, antibiotics, artificial nutrition & hydration, surgery, chemo or radiotherapy, blood transfusion.	MDT approach to symptom control, use of proxies who know patient well, use of symptom assessment tools.	
(Vrijmoeth et al., 2016b)	Physicians' recognition of death in the foreseeable future in patients with intellectual disabilities	Journal of Intellectual Disability Research	Netherlands	Online retrospective survey	To explore when and based on which information sources and signals physicians recognised that a patient with ID would die in the foreseeable future	ID physicians (n=81) & GPs (n=16) who had provided PC to a deceased patient with ID.	All health care settings, Netherlands		Close attention to changes in characteristic behaviour or mood. Gathering information/ collaborating with caregivers & family. Use of tools.	Physician delayed recognition of dying phase in people with ID & delayed discussion of PC until time when death is foreseeable. Diversity in signals that lead to recognition of death. Patient inability to communicate.

(Vrijmoeth et al., 2018)	Development and applicability of a tool for identification of people with intellectual disabilities in need of palliative care (PALLI)	Journal of Applied Research in Intellectual Disabilities	Netherlands	Mixed-methods: online retrospective survey, interviews, focus groups, prospective pilot	To describe the development of PALLI and to explore its applicability as reported by healthcare professionals	**Survey:** GPs (n=16), ID-physicians (n=81). **Interviews:** ID-physicians (n=10), GPs (n=6), DCPs (14). **Focus groups:** ID-physicians (n=11), GPs (n=5), PC nurses for people with ID nurses (n=10), DCPs (n=14). **Pilot:** adults with ID, likely to be in last year of life (n=185).	All health care settings, Netherlands		Use of a tool to identify and assess people with ID who are in need of PC. Includes signals relevant and characteristic to people with ID. Can be completed by physician or carer proxy.	
(Wagemans et al., 2013)	End-of-life decisions for people with intellectual disabilities, an interview study with patient representatives	Palliative Medicine	Netherlands	Qualitative interviews	To clarify the process of end-of-life decision making for people with intellectual disabilities from the perspective of patient representatives.	Family or legal representative of person with ID that had died (n=16)	All health care settings, Netherlands		Doctors closely involved in decision making process, give time to think and deliberate. Building relationships with family.	Over-reliance on family to make complex medical & ethical decisions with limited guidance from professionals. Failure to involve people with IDs themselves in decision making process.
(Wark et al., 2017)	Challenges in providing end-of-life care for people with intellectual disability: Health services access	Journal of Applied Research in Intellectual Disabilities	Australia	Focus group interviews	To explore staff perceptions of the strengths and barriers associated with health service access for people with ID at the end of life	Paid disability support staff who had provided end-of-life support to an individual with an ID (n=35).	4 rural & 2 metropolitan locations, NSW & Queensland		Dr available 24/7. Dr comfortable in supporting people with ID. Community-based health services.	Isolation of staff in rural locations: coping with very complex medical situations with limited resources or advice from health services. Rural patients required to travel long distances to access services. Shortage of rural GPs. Drs refusal to take on patients with ID during the end-of-life: lack of time. Dealing with 'the 'immediate' but no continuity. Diagnostic overshadowing & failure to investigate new health issues. Attitude that using limited resources on people with ID is a waste of money. Drs not prioritizing medical assistance for health issues separate to the existing life-ending condition. Lack of training amongst community & hospital HCPs. Being put in the "too-hard basket." Lack of funding to support additional healthcare needs at EOL. Lack of staff, additional hours required, other residents overlooked to meet the healthcare demands of the dying. Staff fear of caring for the dying: preference to transfer to hospital/NH. Staff stress, recruitment & retention.
(Welch and Moreno-Leguizamon, 2016)	Literature review of best health and social care practices for individuals with intellectual disabilities encountering the end of life	Nursing and Palliative Care	England	Literature review	To identity what were care best practices for individuals with ID encountering the end of life.	Individuals with ID at end of life and those caring for them	All health & social care settings	Culturally competent care. Hydration/nutrition. Pain. Grief & loss. Information. Autonomy.	Training courses, training manuals. Management support. Working strategically & collaboratively with other professionals, within & between organisations. Collaboration with family. Use of communication tools, non-verbal cues, adjusting language. Continuity. Building relationships. Respecting	Lack of policies and procedures governing end-of-life care decision making processes. EOL care provided by mainstream services that are not developed to meet requirements of people with ID. Lack of staff knowledge/experience, lack of time. Staff stress, bereavement. Organisational policy & practice preventing individually-styled approached to communication.

									autonomy. Integrating views of service users into models of care. Time/patience/flexibility. Evidence-based care.	Communication difficulties with patient: ascertaining information requirements, wishes, symptoms.

Abbreviations:
ACP=advance care planning, BBN=breaking bad news, Ca=cancer, CLDT=community learning disability team, CP=cerebral palsy, DCPs=daily care professionals, DNR=do not resuscitate, DNs=district nurses, ED=emergency department, EOL=end of life, F=female, GSF=gold standards framework, H@H=hospice at home, HCA=health care assistants, HCP=health care professionals, ID=intellectual disability, info=information, M=male, MDT=multidisciplinary team, MM=mixed methods, NH=nursing home, NHL=Non-Hodgkin Lymphoma, PC=palliative care, PPD=preferred place of death, Pts=patients, SPC=specialist palliative care

Appendix 3: Hawker et al's critical appraisal tool

---

1. Abstract and title: Did they provide a clear description of the study?
   - Good — Structured abstract with full information and clear title.
   - Fair — Abstract with most of the information.
   - Poor — Inadequate abstract.
   - Very Poor — No abstract.
2. Introduction and aims: Was there a good background and clear statement of the aims of the research?
   - Good — Full but concise background to discussion/study containing up-to-date literature review and highlighting gaps in knowledge. Clear statement of aim AND objectives including research questions.
   - Fair — Some background and literature review. Research questions outlined.
   - Poor — Some background but no aim/objectives/questions OR Aims/objectives but inadequate background.
   - Very Poor — No mention of aims/objectives. No background or literature review.
3. Method and data: Is the method appropriate and clearly explained?
   - Good — Method is appropriate and described clearly (e.g., questionnaires included). Clear details of the data collection and recording.
   - Fair — Method appropriate, description could be better. Data described.
   - Poor — Questionable whether method is appropriate. Method described inadequately. Little description of data.
   - Very Poor — No mention of method, AND/OR Method inappropriate, AND/OR No details of data.
4. Sampling: Was the sampling strategy appropriate to address the aims?
   - Good — Details (age/gender/race/context) of who was studied and how they were recruited. Why this group was targeted. The sample size was justified for the study. Response rates shown and explained.
   - Fair — Sample size justified. Most information given, but some missing.
   - Poor — Sampling mentioned but few descriptive details.
   - Very Poor — No details of sample.
5. Data analysis: Was the description of the data analysis sufficiently rigorous?
   - Good — Clear description of how analysis was done. Qualitative studies: Description of how themes derived/respondent validation or triangulation. Quantitative studies: Reasons for tests selected hypothesis driven/numbers add up/statistical significance discussed.
   - Fair — Qualitative: Descriptive discussion of analysis.

Quantitative.
Poor Minimal details about analysis.
Very Poor No discussion of analysis.

6. Ethics and bias: Have ethical issues been addressed, and what has necessary ethical approval gained? Has the relationship between researchers and participants been adequately considered?
Good Ethics: Where necessary issues of confidentiality, sensitivity, and consent were addressed.
Bias: Researcher was reflexive and/or aware of own bias.
Fair Lip service was paid to above (i.e., these issues were acknowledged).
Poor Brief mention of issues.
Very Poor No mention of issues.

7. Results: Is there a clear statement of the findings?
Good Findings explicit, easy to understand, and in logical progression.
Tables, if present, are explained in text.
Results relate directly to aims.
Sufficient data are presented to support findings.
Fair Findings mentioned but more explanation could be given.
Data presented relate directly to results.
Poor Findings presented haphazardly, not explained, and do not progress logically from results.
Very Poor Findings not mentioned or do not relate to aims.

8. Transferability or generalizability: Are the findings of this study transferable (generalizable) to a wider population?
Good Context and setting of the study is described sufficiently to allow
comparison with other contexts and settings, plus high score in Question 4 (sampling).
Fair Some context and setting described, but more needed to replicate
or compare the study with others, PLUS fair score or higher in Question 4.
Poor Minimal description of context/setting.
Very Poor No description of context/setting.

9. Implications and usefulness: How important are these findings to policy and practice?
Good Contributes something new and/or different in terms of
understanding/insight or perspective.
Suggests ideas for further research.
Suggests implications for policy and/or practice.
Fair Two of the above (state what is missing in comments).
Poor Only one of the above.
Very Poor None of the above.

Appendix 4: Grading of quality

Item score:
Good = 4, Fair = 3, Poor = 2, Very Poor = 1

Total score:
18 or less = poor
19 to 27 = moderate
> 27 = good

**Hawker's 9 item checklist:**	1. Abstract and title: Did they provide a clear description of the study?	2. Introduction and aims: Was there a good background and clear statement of the aims of the research?	3. Method and data: Is the method appropriate and clearly explained?	4. Sampling: Was the sampling strategy appropriate to address the aims?	5. Data analysis: Was the description of the data analysis sufficiently rigorous?	6. Ethics and bias: Have ethical issues been addressed, and what has necessary ethical approval gained? Has the relationship between researchers and participants been adequately considered?	7. Results: Is there a clear statement of the findings?	8. Transferability or generalizability: Are the findings of this study transferable (generalizable) to a wider population?	9. Implications and usefulness: How important are these findings to policy and practice?	**Total**	**Overall grade**
Bailey et al. 2016	3	4	4	2	4	3	4	3	3	**30**	**Good**
Bekkema et al. 2014a	3	4	3	2	4	2	3	3	3	**27**	**Moderate**
Bekkema et al. 2014b	4	3	4	3	4	3	4	3	3	**31**	**Good**
Botsford 2004	3	3	3	2	2	2	3	2	3	**23**	**Moderate**
Brown et al. 2003	2	2	2	3	2	2	3	3	2	**21**	**Moderate**
Cartlidge 2010	4	4	4	3	3	4	3	3	3	**31**	**Good**
Codling et al 2014	3	4	3	3	3	1	3	3	3	**26**	**Moderate**
Cross et al. 2012	3	3	2	2	2	1	3	3	3	**22**	**Moderate**
Dunkley & Sales 2014	3	3	4	4	3	3	4	4	4	**32**	**Good**
Flynn et al 2015	3	3	4	3	3	3	3	2	2	**26**	**Moderate**
Forrester-Jones et al. 2017	4	3	4	3	4	4	3	3	3	**31**	**Good**
Friedman et al 2012	3	3	3	3	3	3	3	4	3	**28**	**Good**
Gilbert et al. 2007	1	1	3	2	2	2	3	2	2	**18**	**Poor**
Grindrod & Rumbold 2017	4	3	3	3	4	2	4	3	4	**30**	**Good**
Hunt et al 2019	4	4	4	4	4	1	4	3	3	**31**	**Good**
Kim & Gray 2018	4	3	3	3	4	4	4	2	2	**29**	**Good**
Kinley et al 2018	4	3	3	2	2	1	3	2	3	**23**	**Moderate**
Lindop & Read 2000	3	4	3	3	3	1	2	2	3	**24**	**Moderate**

Marriott et al 2013	4	3	3	2	1	1	2	1	2	**19**	**Moderate**
McKechnie 2006	3	2	1	1	1	1	2	2	2	**15**	**Poor**
McLaughlin et al. 2014	4	3	4	3	4	3	3	3	3	**30**	**Good**
McNamara et al. 2019	4	3	3	3	3	3	4	3	3	**29**	**Good**
Moro et al. 2017	4	4	3	4	3	2	3	3	3	**29**	**Good**
Morton-Nance & Schafer 2012	4	4	4	2	3	3	3	2	2	**27**	**Moderate**
Ng & Li 2003	3	3	3	3	3	3	3	2	3	**26**	**Moderate**
Northway et al. 2019	4	3	3	3	3	2	3	2	3	**26**	**Moderate**
O'Regan & Drummond 2008	3	2	2	2	2	2	3	3	3	**22**	**Moderate**
O'Sullivan & Harding 2017	4	3	3	2	4	3	3	2	3	**27**	**Moderate**
Read & Cartlidge 2012	4	3	3	2	2	4	3	2	2	**25**	**Moderate**
Reddall 2010	3	3	2	2	2	1	3	2	2	**20**	**Moderate**
Ronneberg et al 2015	2	1	2	2	2	1	3	2	2	**17**	**Poor**
Ryan et al 2016	4	3	3	2	3	1	3	2	3	**24**	**Moderate**
Ryan & McQuillan 2005	3	2	1	1	1	1	3	2	2	**16**	**Poor**
Sampson et al. 2015	4	4	3	3	3	1	3	3	3	**27**	**Moderate**
Stein 2008	3	2	3	2	2	1	3	2	3	**21**	**Moderate**
Sue et al 2019	3	2	2	3	2	1	3	3	3	**22**	**Moderate**
Tuffrey-Wijne 2003	4	3	3	3	3	2	3	3	3	**27**	**Moderate**
Tuffrey-Wijne et al. 2007 (a)	4	3	4	3	3	3	3	3	3	**29**	**Good**
Tuffrey-Wijne et al. 2007 (b)	4	3	3	2	3	3	3	2	3	**26**	**Moderate**
Tuffrey-Wijne et al. 2008	4	3	3	3	3	3	3	3	3	**28**	**Good**
Tuffrey-Wijne & McEnhill 2008	4	3	2	2	1	1	3	2	3	**21**	**Moderate**
Tuffrey-Wijne et al. 2009	4	3	4	4	4	2	3	3	3	**30**	**Good**
Tuffrey-Wijne 2013	4	4	3	3	4	4	3	3	3	**31**	**Good**

Tuffrey-Wijne et al. 2016	4	3	4	4	3	3	4	3	4	**32**	**Good**
Tuffrey-Wijne et al 2017	4	4	3	2	3	3	3	3	3	**28**	**Good**
Tuffrey-Wijne & Davidson 2018	4	3	4	4	3	3	4	2	3	**30**	**Good**
Vrijmoeth et al. 2016 (a)	4	3	4	3	4	1	4	3	3	**29**	**Good**
Vrijmoeth et al 2016 (b)	4	3	3	3	3	3	3	2	3	**27**	**Moderate**
Vrijmoeth et al 2018	4	3	4	3	3	3	3	2	3	**28**	**Good**
Wagemans et al. 2013	4	3	4	3	4	2	3	2	3	**28**	**Good**
Wark et al. 2017	4	2	3	2	3	3	3	2	3	**25**	**Moderate**
Welch & Moreno-Leguizamon 2016	3	3	4	4	3	3	3	3	3	**29**	**Good**
